# Occurrence and Multilocus Genotyping of* Giardia duodenalis* in Yunnan Black Goats in China

**DOI:** 10.1155/2018/4601737

**Published:** 2018-10-10

**Authors:** Shi-Chen Xie, Yang Zou, Dan Chen, Meng-Meng Jiang, Xiao-Dan Yuan, Zhao Li, Feng-Cai Zou, Jian-Fa Yang, Jin-Liang Sheng, Xing-Quan Zhu

**Affiliations:** ^1^College of Animal Science and Technology, Shihezi University, Shihezi, Xinjiang Uygur Autonomous Region 832003, China; ^2^State Key Laboratory of Veterinary Etiological Biology, Key Laboratory of Veterinary Parasitology of Gansu Province, Lanzhou Veterinary Research Institute, Chinese Academy of Agricultural Sciences, Lanzhou, Gansu Province 730046, China; ^3^Key Laboratory of Veterinary Public Health of Yunnan Province, College of Veterinary Medicine, Yunnan Agricultural University, Kunming, Yunnan Province 650201, China; ^4^Jiangsu Co-innovation Center for the Prevention and Control of Important Animal Infectious Diseases and Zoonoses, Yangzhou University College of Veterinary Medicine, Yangzhou, Jiangsu Province 225009, China

## Abstract

*Giardia duodenalis *is an important zoonotic parasite which can parasitize in the intestines of humans and various animals. However, the information about the prevalence and genetic diversity of* G. duodenalis* in goats in China is limited. It is yet to be known whether Yunnan black goats, a unique goat breed in subtropical Yunnan province, southwestern China, are infected with* G. duodenalis.* Thus, a total of 907 fecal samples were collected from Yunnan black goats in five regions in Yunnan province, to estimate the prevalence and genotypes of* G. duodenalis* using a PCR-based approach. The* G. duodenalis* prevalence is 4.2% (38/907) in Yunnan black goats by nested amplification of the *β*-giardin (bg) gene, and the genotypes are identified as assemblage E, with 5 novel subtypes (E_11_-E_15_). Multilocus sequence typing revealed that 11, 18, and 38 samples were amplifiable on tpi (triose phosphate isomerase), gdh (glutamate dehydrogenase), and bg locus, respectively, and identified three novel multilocus genotypes (MLGs): MLGE9-MLGE11. To our knowledge, this is the first report of* G. duodenalis* prevalence and genotypes in Yunnan black goats in China, which extended the host range of* G. duodenalis* and provided basic data for controlling* G. duodenalis* infection in Yunnan black goats.

## 1. Introduction

*Giardia duodenalis* (syn.* Giardia lamblia* and* Giardia intestinalis*) is a common enteric protozoan parasite which can infect humans and a wide range of animal species.* G. duodenalis* infection can cause a series of diseases which have important effects on human and animal health, such as abdominal cramps, diarrhea, weight loss, and malabsorption [1–3].* G. duodenalis* infection can be caused by ingesting cysts in contaminated water or food, or through fecal-oral access due to wastewater [4–6]. According to the existing literature, the prevalence of* G. duodenalis* is approximately 10% in the world population [7], and the* G. duodenalis* prevalence ranged from 0 to 15.6% in humans [8, 9] and 1.3%-55.6% in sheep and goats in China [1].* G. duodenalis* has a high prevalence in some low-income areas and developing countries [10–13].

So far,* G. duodenalis* isolates from humans and various animals have been classified into eight different assemblages (A-H) on the basis of molecular characterization [14, 15]. Among them, assemblages A and B are the important zoonotic parasites that have a wild range of hosts, including human and other mammals, such as bovine, sheep, goats, and other domestic animals [1, 3]. Assemblage E occurs in artiodactyls, and assemblages C, D, F, G, and H have obvious animal specificity, but assemblages C-F have also been reported in humans in Ethiopia [16], Thailand [17], and Egypt [18].

Yunnan province is the fifth largest producer of goats in China [19], and about 10 million goats are raised each year. Many previous studies have reported* G. duodenalis* infection in goats in other countries with prevalence ranging from 2.9 to 35.8% [20, 21], but only limited investigations have been conducted in goats in China, with the prevalence ranging from 2.9 to 7.1% [22–25].

Yunnan black goat is a unique breed of goat distributed in subtropical Yunnan province, southwestern China. It is yet to be known whether Yunnan black goats are infected with* G. duodenalis*. Thus, the objectives of the present study were to estimate the* G. duodenalis *prevalence in Yunnan black goats based on characterization of the *β*-giardin (*bg*) gene sequences and identify its genotypes using multilocus genotyping (MLG) targeting gdh gene, tpi gene, and bg gene sequences [15, 26].

## 2. Materials and Methods

### 2.1. Animals and Samples Collection

**Figure 1:**
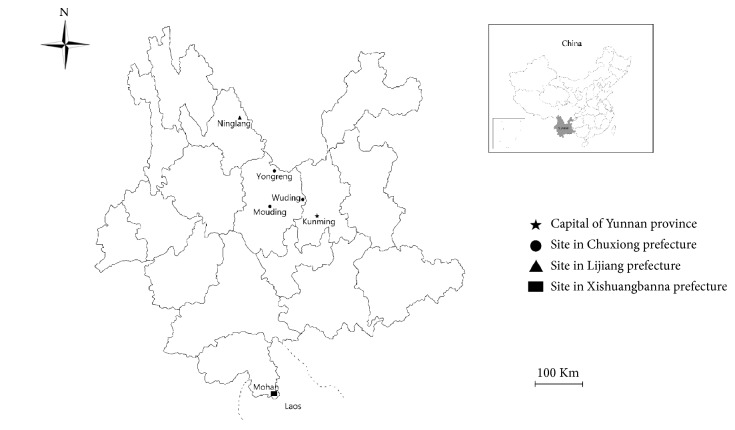
A map depicting the sampling sites for collecting fecal samples from Yunnan black goats in Yunnan province, southwestern China.

A total of 907 fecal samples were randomly collected from Yunnan black goats in Chuxiong, Lijiang, and Xishuangbanna prefectures, Yunnan province, southwestern China (Figure 1). All of the fecal samples were stored in separate sterile plastic collection tubes containing 2.5% potassium dichromate, kept cold with ice packs, transported to the laboratory as soon as possible, and kept in 4°C freezer until analysis. The sample information including geographical gender, age, locality, and date of sampling was recorded.

### 2.2. Genomic DNA Extraction

Fecal specimens were washed repeatedly with ultrapure water until all the potassium dichromate was removed, and then genomic DNA was extracted from 200 mg of each fecal sample in a 2 ml centrifuge tube using the commercial E.Z.N.A® Stool DNA kit (Omega Bio-Tek Inc., GA, USA) by following the manufacturer's instruction. The obtained DNA samples were stored at −20°C for further study.

### 2.3. PCR Amplification and Sequencing

Each fecal specimen was examined for the presence and genotype of* G. duodenalis* by PCR-based sequencing of the 511 bp fragment of the bg-gene [27]. In addition, For MLG analysis, all bg-positive specimens were subjected to further PCR using primers for the tpi gene loci and gdh gene loci [28–30]. The sequences of primers are presented in Table 1.

The secondary reaction mixture contained 2 *μ*l of template from the first PCR product, 2 *μ*L deoxyribonucleotide triphosphate (dNTP) mixture, 2.5 *μ*L of 10×PCR buffer, 3 mM of MgCl_2_, and 0.2 *μ*M of each primer in a total volume of 25 *μ*L. PCR amplifications were performed as follows: 1 cycle for 5 min at 94°C, followed by 35 cycles of 45 s at 94°C for denaturation, 45 s at 67°C for annealing, and 45 s at 72°C for an extension. All of amplification products were subsequently visualized on 1.5% agarose gels with ethidium bromide. For each PCR amplification, a positive sample (sequenced DNA) and negative (PCR water) control sample were included.

All nested-PCR products were sent to Xi'an Qingk Biotechnology Company for two-directional sequencing on an ABI PRISM 3730 XL DNA Analyzer (Applied Biosystems, Foster City, CA, USA) using relevant internal nested primers for PCR amplification. The sequences obtained were compared with relevant sequences available in GenBank database (http://www.ncbi.nlm.nih.gov/GenBank) using Basic Local Alignment Search Tool (BLAST).

### 2.4. Phylogenetic Analysis

The tpi gene sequences were used for phylogenetic reconstruction using the Neighbor-Joining [NJ] analysis and the genetic distances were calculated by the Kimura 2-parameter model in MEGA6 [31, 32]. Bootstrap analysis (1000 replicates) was used to evaluate the reliability of the phylogenetic tree [33].

### 2.5. Statistical Analysis

The relationships between* G. duodenalis *prevalence and risk factors were analyzed using the* x*^*2*^ test in SPSS 20.0 (SPSS Inc., Chicago, IL, USA), and statistically significant differences were considered when* P* <0.05.

## 3. Results and Discussion

### 3.1. The Prevalence of G. duodenalis in Yunnan Black Goats

A total of 907 fecal samples were collected from Yunnan black goats in five regions in Yunnan province (Figure 1), and 38 (4.2%, 95% CI, 2.9-5.5) were* G. duodenalis*-positive based on the amplification of the bg gene.* G. duodenalis* prevalence was significantly different among the study areas (*χ*2=10.933,* df*=4,* P *< 0.05), between different age groups (*χ*2=5.208,* df*=1,* P* < 0.05), and between different genders (*χ*2=1.615,* df*=1,* P* > 0.05). The* G. duodenalis* prevalence in Yunnan black goats was higher than that (2.9%) in goats in Heilongjiang province [22], but lower than that in goats in Anhui (6.3%) [23], Shaanxi (7.9%) [25], and Henan provinces (12.7%) [24], China. The* G. duodenalis* prevalence in Yunnan black goats was markedly lower than in goats in Greece (40.4%) [34], Spain (42.0%) [20], Uganda (40.7%) [35], and Belgium (35.8%) [21]. The difference in* G. duodenalis* prevalence may be related to feeding conditions, geographical difference, and animal husbandry practices as well as different susceptibility of different breeds of goats.

*G. duodenalis* prevalence ranged from 0% to 7.03% among the sampled areas. The highest* G. duodenalis* prevalence was found in Yunnan black goats in Mohan (7.03%, 9/128), Xishuangbanna prefecture (Table 2), followed by Wuding (5.41%, 24/444) in Chuxiong prefecture, Ninglang (1.96%, 1/51) in Lijiang prefecture, and Yongreng (1.43%, 2/139) and Mouding (1.38%, 2/145) in Chuxiong prefecture. The likely reason for this discrepancy may be due to different geographical conditions.

### 3.2. Molecular Characterization of G. duodenalis Isolates

All the bg sequences obtained in the present study were aligned with corresponding* G. duodenalis* sequences available in GenBank by BLAST. A total of 38 positive samples were clustered in assemblage E, containing one known assemblage E subtype (E_5_, n=35) and two novel assemblage E subtypes (designated as E_14_, n=1; E_15_, n=2) based on sequence analyses of the bg gene loci (Table 3). Additionally, one known assemblage E subtype (E_10_, n=2) and one novel assemblage E subtype (E_13_, n=16) based on the gdh gene sequences and two novel assemblage E subtypes (E_11_, n=1; E_12_, n=10) based on the tpi gene sequences were also identified among* G. duodenalis*-positive samples from Yunnan black goats (Table 3).

Previous studies have indicated that assemblage E is the predominant genotype infecting a range of hoofed livestock; it is also the most common assemblage found in sheep, goats, and pigs. However, assemblage E has also been identified in cattle, dogs, cats, horses, fallow deer, monkeys, and humans [1, 3, 29, 36] indicating that assemblage E is of zoonotic significance.

MLG analysis based on bg, gdh, and tpi gene sequences is a useful tool to illustrate the diversity of the* G. duodenalis* genotypes [37]. In this study, 18 of the 38 bg-positive samples were gdh-positive, and 11 were tpi-positive. Ten samples were successfully sequenced at all of the three loci, and three novel MLGs (designated as MLGE9-E11) were identified within assemblage E (Table 4).

### 3.3. Phylogenetic Analysis of G. duodenalis Isolates from Yunnan Black Goats

**Figure 2:**
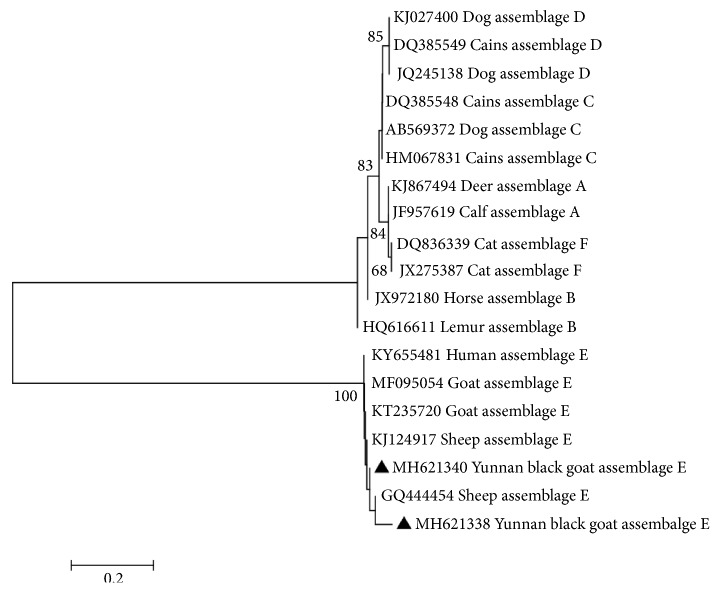
The phylogenetic relationships among* G. duodenalis *isolates inferred by a Neighbor-Joining (NJ) algorithm using a Kimura two-parameter analysis (1000 replicates) based on the tpi gene sequences. The two novel assemblage E subtypes E_11_ and E_12_ (MH621338, MH621340) are marked by filled triangles.

To clarify the genetic relationships of the* G. duodenalis* isolates in this study with relevant* G. duodenalis *isolates, the obtained* G. duodenalis* tpi gene sequences were aligned with corresponding sequences available in the GenBank database. The phylogenetic tree showed that* G. duodenalis *isolates from Yunnan black goats clustered within assemblage E which contained* G. duodenalis *isolates (E_11_ and E_12_) from other animals and humans (Figure 2), with a high bootstrap value, indicating that* G. duodenalis* genotypes in Yunnan black goats have zoonotic potential, raising a public health concern.

## 4. Conclusion

This is the first report of prevalence and molecular characterization of* G. duodenalis* from Yunnan black goats in Yunnan province, southwestern China, which revealed a 4.2%* G. duodenalis *prevalence and identified seven subtypes including five novel assemblages E subtypes (E_11_-E_15_) and two known assemblages E subtypes (E_5_ and E_10_). MLGs analysis identified three novel MLGs within assemblage E of* G. duodenalis. *These results not only extended the host range of* G. duodenalis* distribution, but also enriched the genetic diversity of* G. duodenalis* in humans and animals, which also have implications for controlling* G. duodenalis* infection in Yunnan black goats.

## Figures and Tables

**Table 1:** Primers used in the study; annealing temperatures used in the PCRs.

Gene	Primer	Sequences (5′-3′)	Annealing temperature (°C)	Reference
bg	GF1	AAGCCCGACGACCTCACCCGCAGTGC	55	[1]
	GR1	GAGGCCGCCCTGGATCTTCGAGACGAC		
	GF2	GAACGAACGAGATCGAGGTCCG	55	
	GR2	CTCGACGAGCTTCGTGTT		
gdh	Gdh1	TTCCGTRTYCAGTACAACTC	50	[1]
	Gdh2	ACCTCGTTCTGRGTGGCGCA		
	Gdh3	ATGACYGAGCTYCAGAGGCACGT	65	
	Gdh4	GTGGCGCARGGCATGATGCA		
tpi	AL3543	AAATIATGCCTGCTCGTCG	50	[1, 3]
	AL3546	CAA ACCTTITCCGCAAACC		
	ALEf	CCCCTTCTGCCGTACATTTAT	58	
	ALEr	GGCTCGTAAGCAATAACGACTT		

**Table 2:** Prevalence and risk factors of *Giardia duodenalis* infection in Yunnan black goats in Yunnan province, southwestern China.

Factor	Category	No. tested	No. positive (%) [95% CI]	OR [95 % CI]	*P*-value
Area	Wuding	444	24 (5.4, 3.3-7.5)	4.086 (0.95-17.50)	0.04
	Yongreng	139	2 (1.4, 0.6-3.3)	1.044 (0.15-7.51)	0.97
	Mouding	145	2 (1.4, 0.5-3.3)	Ref	Ref
	Ninglang	51	1 (2.0, 1.8-5.8)	1.430 (0.13-16.11)	0.77
	Mohan	128	9 (7.0, 2.6-11.5)	5.408 (1.15-25.51)	0.02
Gender	Female	633	23 (3.6, 2.1-5.1)	0.651 (0.33-1.27)	0.20
	Male	274	15 (5.5, 2.8-8.2)		
Age (month)	≤12	364	22 (6.1, 3.6-8.6)	2.119 (1.10-4.09)	0.02
	>12	543	16 (2.9, 1.2-4.6)		
Total		907	38 (4.2, 2.9-5.5)		

**Table 3:** Intra-assemblage substitutions in tpi, gdh, and bg loci within *Giardia duodenalis* assemblage E.

Subtypes (number)	Nucleotide position and substitutions					GenBank ID
tpi						
	188	248				
Ref. sequence	G	A				MF095054
E_11_ (1)	C	T				MH621338
E_12_ (10)	G	A				MH621340
gdh						
	391	608	623			
Ref. sequence	C	A	A			KX813711
E_10_ (2)	T	G	G			
E_13_ (16)	C	G	G			MH621339
bg						
	62	66	78	82	365	
Ref. sequence	C	A	A	T	C	KY769092
E_5_ (35)	C	A	A	T	C	
E_14_ (1)	A	-	G	G	C	MH621337
E_15_ (2)	C	A	A	T	T	MH621341

**Table 4:** Multilocus characterization of *Giardia duodenalis* isolates based on the tpi, gdh, and bg genes.

subtype			No. of sequences	MLG type
tpi	gdh	bg		
E_12_	E_13_	E_15_	1	MLGE9
E_12_	E_13_	E_5_^a^	8	MLGE10
E_11_	E_13_	E_5_^a^	1	MLGE11
-	E_13_	E_15_	1	
-	E_10_^b^	E_5_^a^	2	
-	E_13_	E_5_^a^	5	
E_12_	-	E_5_^a^	1	
-	-	E_14_	1	
-	-	E_5_^a^	18	

Note: a, b indicate that genotypes have been reported.

-: not determined.

## Data Availability

The* Giardia duodenalis* prevalence data used to support the findings of this study are included within the article.
